# DNA binding reduces the dissociation rate of STAT1 dimers and impairs the interdimeric exchange of protomers

**DOI:** 10.1186/s12858-014-0028-z

**Published:** 2014-12-20

**Authors:** Theresa Riebeling, Julia Staab, Christoph Herrmann-Lingen, Thomas Meyer

**Affiliations:** Klinik für Psychosomatische Medizin und Psychotherapie, Georg-August-Universität Göttingen, Waldweg 33, 37073 Göttingen, Germany

**Keywords:** STAT, Dimerization, Interferon signalling, Transcriptional activation

## Abstract

**Background:**

A shift between two dimer conformations has been proposed for the transcription factor STAT1 (*s*ignal *t*ransducer and *a*ctivator of *t*ranscription 1) which links DNA binding of the parallel dimer to tyrosine dephosphorylation of the antiparallel dimer as two consecutive and important steps in interferon- γ (IFNγ)-mediated signalling. However, neither the kinetics nor the molecular mechanisms involved in this conformational transition have been determined so far.

**Results:**

Our results demonstrated that the dissociation of dimers into monomers and their subsequent re-association into newly formed tyrosine-phosphorylated dimers is a relatively slow process as compared to the fast release from high-affinity DNA-binding sites, termed GAS (gamma-activated sequence). In addition, we noted an inhibitory effect of GAS binding on the exchange rate of protomers, indicating that DNA binding substantially impedes the recombination of dimeric STAT1. Furthermore, we found that reciprocal aminoterminal interactions between two STAT1 molecules are not required for the interchange of protomers, as an oligomerization-deficient point mutant displayed similar interdimeric exchange kinetics as the wild-type molecule.

**Conclusions:**

Our results demonstrate that DNA binding impairs the oscillation rate between STAT1 conformers. Furthermore, these data suggest that the rapid release from high-affinity GAS sites is not a rate-limiting step in IFNγ-mediated signal transduction. Further investigations are needed to decipher the physiological significance of the observed dissociation/re-association process of STAT1 dimers.

## Background

A variety of transcription factors function as dimeric proteins that recognize dyadic and palindromic DNA sequences in promoter regions and facilitate gene expression by recruiting the transcription initiation complex including RNA polymerase II. Sequence-specific DNA binding of dimeric transcription factors requires the presence of two half-sites localized in a specific orientation and spacing so that each protomer can interact with the DNA-binding motif [1]. The binding topography for each protomer in a dimeric transcription factor bound to DNA is the same in the sense that there are two identical and non-overlapping protein-DNA contacts forming between each protomer and the DNA double helix. While dimerization of signal-dependent transcription factors is often a prerequisite for optimal DNA binding to natural response elements, some transcriptional regulatory proteins also recognize DNA binding sites as monomers.

Homodimeric transcription factors recognizing such small DNA-sequence motifs are typically grouped into families on the basis of structural homology in their dimerization and DNA-binding properties. The family of STAT (*s*ignal *t*ransducer and *a*ctivator of *t*ranscription) proteins serves the dual function of signal transduction and transcriptional activation in cytokine signalling. In humans, seven STAT genes have been identified, and their encoded proteins are engaged in such diverse cellular processes as growth control, proliferation, differentiation, and immune response [2]. The founding member of this family, STAT1, is well characterized for its role in transmitting interferon signals from membrane-bound cytokine receptors to the nucleus where it drives transcription. Interferon-γ (IFNγ)-induced nuclear accumulation of STAT1 homodimers is a hallmark of this signal pathway which requires binding of the cytokine to its cognate receptor and the auto-phosphorylation of non-covalently receptor-associated Janus kinases (JAKs) [3,4]. The activated JAK kinases then phosphorylate the intracellular receptor tail, thereby creating phospho-tyrosine docking sites for the recruitment of STAT1 via its Src-homology-2-(SH2) domain. Subsequently, JAKs phosphorylate a signature tyrosine residue (Y701) in the carboxyterminus of STAT1 which results in the formation of parallel STAT1 homodimers stabilized through reciprocal SH2-phosphotyrosine interactions [4-8]. The tyrosine-phosphorylated dimers are then transported via a Ran-dependent, importin-α/β-mediated transport pathway to the nucleus. There they bind to palindromic GAS (gamma-activated site) elements in the promoter regions of IFNγ-responsive genes [9-12]. The consensus GAS motif 5′-TTC(N)_3-4_GAA-′3 has been found in sequences recognized by most members of the STAT family [13,14]. After dissociation from DNA, STAT1 dimers are dephosphorylated by the nuclear tyrosine phosphatase Tc45 which is a prerequisite for their nuclear export [15-18]. The cytoplasmic STAT1 proteins may then be rephosphorylated depending on receptor occupancy and participate in another cycle of nucleocytoplasmic shuttling. In addition to this carrier-mediated nuclear import of phospho-STAT1 in IFNγ-stimulated cells, a highly dynamic translocation between the cytosolic and nuclear compartment was uncovered also for unphosphorylated STAT1 in resting cells which is mediated through direct contacts of residues in the STAT1 linker domain with nucleoporins located in the nuclear pore complex [19,20].

**Figure 1:**
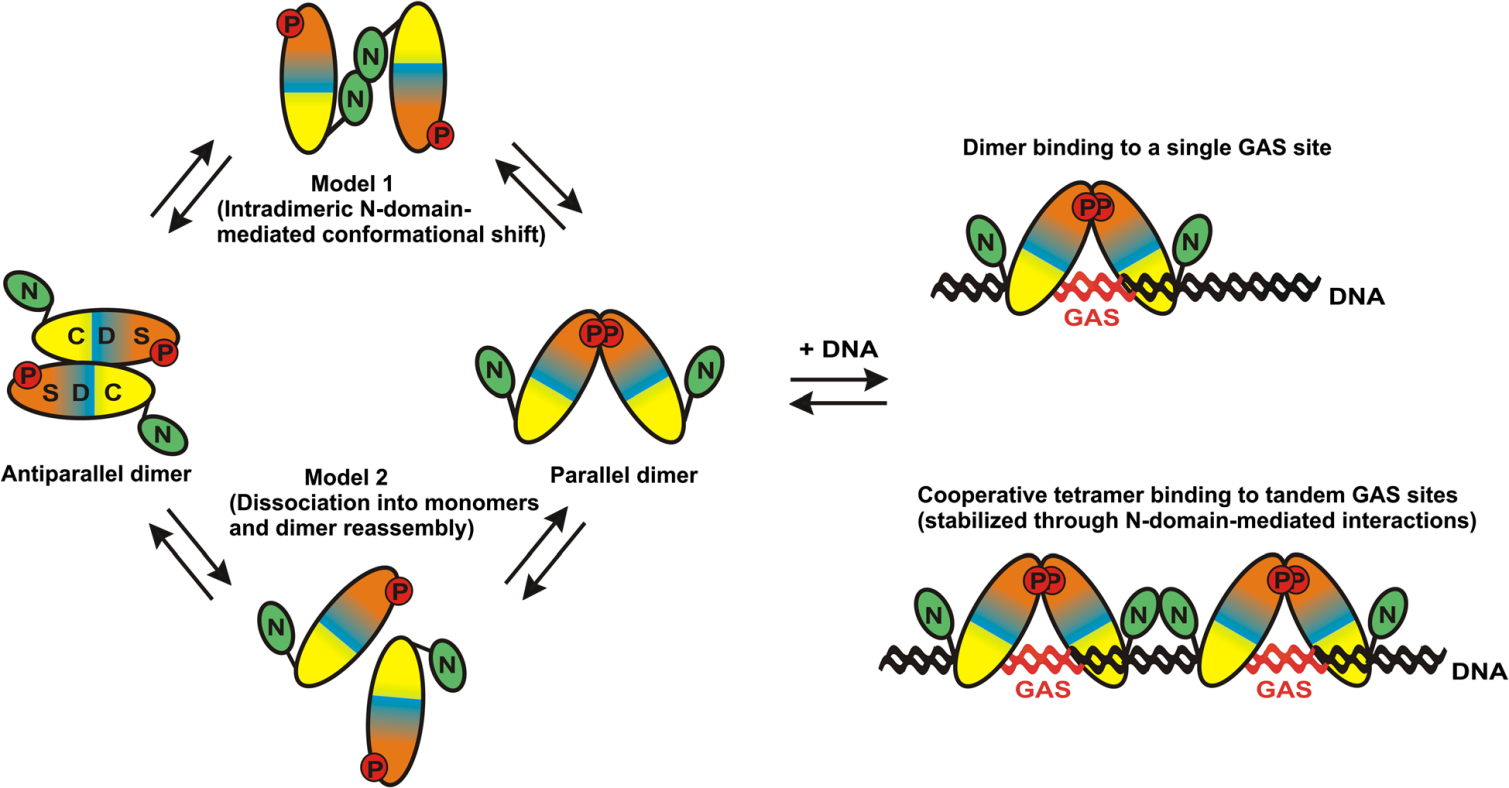
**Graphic illustration of putative pathways for the interconversion of parallel and antiparallel STAT1 conformers.** The domain structure of STAT1 is marked with different colours: the N-terminal domain (N, green) is added via a short flexible segment to the core fragment comprising the coiled-coil domain (C, yellow), the DNA-binding domain (D, blue), the linker domain (not shown), the SH2 domain (S, orange), and the carboxyterminal transactivation domain (not shown). The interface in the antiparallel dimer is formed by reciprocal binding between the coiled-coil domain of one protomer and the DNA-binding domain of the partner protomer, while in the parallel dimer there are reciprocal interactions between the phospho-tyrosine residue 701 (P, marked as red circles) and the SH2 domain. Model 1 (top left) assumes that the core domains of the two partner protomers rotate around each other facilitated by reciprocal N-terminal interactions [21,22]. In contrast, the dissociation/re-association model (model 2, bottom left) allows the formation of new dimer combinations and does not require the presence of the N-terminal domain [23]. Addition of specific DNA-binding elements termed gamma-activated sites (GAS, marked in red) results in the formation of STAT1 dimers bound to a single GAS site (top right) or STAT1 tetramers when complexed with two GAS sites arranged in a tandem orientation (bottom right).

Previous studies have proposed that dephosphorylation of tyrosine residue 701 requires extensive spatial reorientation of the protomers from a parallel to an antiparallel dimer conformation (Figure 1) [21,22]. Using an extensive mutagenesis approach, Darnell and co-workers have suggested that after phosphotyrosine-SH2 disjunction the phospho-STAT1 dimer is still held together by reciprocal aminoterminal interactions [21,22] (Figure 1). This spatial reorganization from a parallel to an antiparallel dimer conformation might expose the phosphotyrosine residue 701 as a target of the Tc45 phosphatase. However, it remains controversial whether the interconversion of conformers requires that the interacting partner protomers are individually connected to each other. This would most probably occur through the N-domains, while the two core fragments would undergo extensive spatial reorientation (Figure 1). A stretch of amino-acid residues within the coiled-coil domain functions as a linker and ensures that the position of the N-domain in relation to the core fragment is flexible [22]. An alternative model proposes that the STAT1 dimers first dissociate into isolated monomers and then reassembled into new dimeric complexes (Figure 1). In contrast to the first model, this dissociation and re-association cycle allows the formation of recombined dimers. Using analytical ultracentrifugation of purified recombinant STAT1 proteins, the Vinkemeier laboratory revealed that STAT1 constantly oscillated between the two dimer conformations and that both are equally stable with a dissociation constant (K_d_) of approximately 50 nM [23]. In addition, the authors demonstrated that the STAT1 aminoterminal domain is engaged in the formation of high-affinity tetrameric complexes. However, from these ultracentrifugation experiments, neither the molecular mechanisms nor the time course of interdimeric protomer exchange can be resolved. Thus, we performed kinetic measurements by means of gel-shift assays to analyse the stability of dimeric STAT1 complexes *in vitro*.

## Methods

### Cell culture

STAT1-negative U3A cells [24] were cultured in a humidified 5% CO_2_ atmosphere at 37°C in Dulbecco’s modified Eagle’s medium (PAA Laboratories) supplemented with 10% foetal calf serum (FCS), 1% penicillin, and 1% streptomycin (Biochrom). Cells were transfected with the transfection reagent MegaTran1.0 (Origene) according to the manufacturer’s recommendation. Twenty-four hours after transfection, cells were stimulated for 45 min with 50 ng/ml recombinant human IFNγ (Biomol).

### Plasmids

For expression of STAT1 tagged with green-fluorescent protein (GFP) in human cells, we used the plasmid pSTAT1-GFP [25]. Based on the vector pEGFP-N1, it coded for a recombinant full-length human STAT1 protein (amino acids 1–746) fused to GFP in carboxyterminal orientation. For expression of untagged STAT1, U3A cells were transfected with the vector pcDNA3.1 (Invitrogen) coding for full-length human STAT1, which is termed here pSTAT1 [25]. The pSTAT1-GFP and pSTAT1 vectors coded either for wild-type (WT) STAT1 or an aminoterminal point mutant thereof with a substitution of alanine for phenylalanine in position 77 [26]. The F77A mutation in the two plasmids was confirmed by DNA sequencing.

### Protein extraction

Cells expressing either GFP-tagged or untagged STAT1 were cultured on 6-well dishes and on the day after transfection treated for 45 min with 50 ng/ml recombinant human IFNγ. Cells were then lysed for 5 min on ice in 50 μl cytoplasmic extraction buffer containing 20 mM Hepes, pH 7.4, 10 mM KCl, 10% (v/v) glycerol, 1 mM EDTA, 0.1 mM Na_3_VO_4_, 0.1% IGEPAL-CA-360, 3 mM DTT, 0.4 mM Pefabloc, and Complete Mini protease inhibitors (Roche). The lysates were centrifuged at 16000×g (15 sec, 4°C), and supernatants spun again at 4°C for 5 min at 16000×g. The supernatants resulting from this centrifugation step were used as cytoplasmic extracts, while the pellets were resuspended in 50 μl nuclear extraction buffer (20 mM Hepes, pH 7.4, 420 mM KCl, 20% (v/v) glycerol, 1 mM EDTA, 0.1 mM Na_3_VO_4_, 3 mM DTT, 0.4 mM Pefabloc, and Complete Mini protease inhibitors) and left on ice for 30 min. The samples were centrifuged at 16000×g for 15 min (4°C) and the supernatants collected as nuclear extracts. Equal amounts of cytoplasmic and nuclear extracts from the same samples were mixed and the whole cell extracts stored at −80°C until further use.

### Western blotting

For immunoblotting, the combined cytosolic and nuclear lysates were boiled in 6× Laemmli buffer, resolved by 10% SDS-PAGE and blotted onto PVDF membranes. The membranes were incubated first with a rabbit monoclonal antibody directed against tyrosine-phosphorylated STAT1 (Cell Signaling, clone 58D6, #9167) and then with a secondary anti-rabbit antibody conjugated to IRDye 800CW (LI-COR Biosciences, #926-32213). After detection of bound immunoreactivity on a LI-COR Odyssey imaging machine, the blots were stripped for 60 min at 60°C in a buffer containing 2% SDS, 0.7% β-mercaptoethanol, and 62.5 mM Tris–HCl, pH 6.8, and subsequently reprobed with polyclonal antibody C-24 directed against STAT1 (Santa Cruz Biotechnology, #sc-345) and secondary antibody.

### Electrophoretic mobility shift assay

Cellular extracts from IFNγ-stimulated cells expressing either STAT1-GFP or untagged STAT1 were probed for binding activity to DNA containing one or two high-affinity consensus GAS sites [27]. For use in gel-shift experiments, DNA probes were generated by hybridizing inverse, complementary oligonucleotides containing poly-(T)_5_ overhangs on the 5′ end of each primer. In a subsequent end-filling reaction catalysed by the Klenow fragment (New England Biolabs), the hybridized oligonucleotides were incubated with desoxy-ATP [^33^P]-labelled on its alpha-phosphate group (Hartmann Analytic). The sequences of the two duplex oligonucleotides used in this study were as follows (overhangs at the 5′ ends and the respective antisense oligos are not included, half-sites of GAS elements in bold italic): M67; 5′-CGACAT***TTC****CCG****TAA***ATCTG-′3, and 2xGAS; 5′-CGT***TTC****CCC****GAA***ATTGACGGAT***TTC****CCC****GAA***AC-′3. In all electrophoretic mobility shift assays (EMSA), 5 microliters of cellullar extracts reacted with 1 ng of the [^33^P]-labelled duplex oligonucleotide probe.

To assess the dissociation of endogenous STAT1 from a single GAS element, a 750-fold molar excess of unlabelled M67 DNA was incubated with the shift reactions for the indicated times on ice. Similar competition experiments were performed for the identification of STAT1-containing complexes. In addition, supershift reactions were performed at RT by incubating the shift reactions for 30 min with 20 ng of either STAT1-specific C-24 antibody or the unspecific H-190 antibody directed against STAT3 (both from Santa Cruz Biotechnology). For co-incubation experiments, we mixed whole cell extracts from U3A cells exclusively expressing either GFP-tagged or untagged STAT1 and immediately incubated the mixed samples with the radioactively labelled GAS probe for 45 min. In a second approach, we combined the extracts in the absence of the GAS probe, which was added after 45 min immediately before gel electrophoresis. In control experiments, we separately incubated similar amounts of the extracts each with the [^33^P]-labelled probe and combined the reactions not before the 45 min co-incubation period was accomplished. Thus, in the first two experiments but not in the control experiments, GFP-tagged and untagged STAT1 dimers had the chance to recombine and exchange their protomeric components.

### Statistical analyses

Binding intensities on autoradiographs were quantified using the ImageJ software from at least triplicate gel-shift experiments. Means and standard deviations were calculated for each STAT1-containing complex bound to DNA. Differences in DNA-binding activity were analyzed by ANOVA with Tukey’s post-hoc tests. All data were analysed using the Sigmastat (Systat Software) program. In all analyses, a p value ≤0.05 was used to indicate statistical significance.

## Results

### Fusion with GFP did not affect the DNA-binding kinetics of STAT1

**Figure 2:**
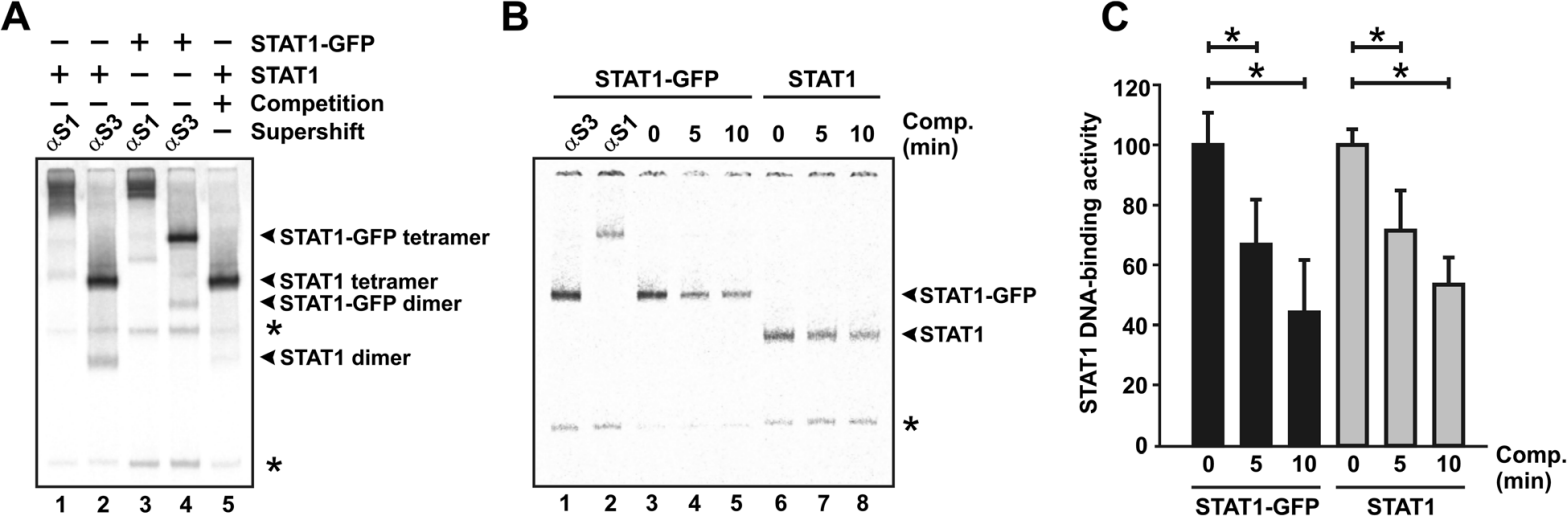
**(A) Electrophoretic mobility shift assay (EMSA) for the identification of green-fluorescent protein-tagged STAT1 (STAT1-GFP) and untagged STAT1.** Extracts from reconstituted STAT1-negative U3A cells expressing recombinant GFP-tagged or untagged STAT1 were incubated at room temperature with a [^33^P]-labelled double-stranded oligonucleotide containing two GAS sites in tandem orientation (2xGAS). Supershift reactions were performed by adding anti-STAT1 antibody C-24 (lanes 1 and 3), and, as control, an unspecific STAT3 antibody (lanes 2 and 4). For competition, a 750-fold molar excess of unlabelled GAS was added to the reaction (lane 5). Asterisks mark unspecific bands. **(B,C)** Similar dissociation kinetics of STAT1-GFP and STAT1 from a single consensus GAS site (M67). Cellular extracts from U3A cells expressing tagged or untagged STAT1 were incubated for 15 min with [^33^P]-labelled M67 before on ice a 750-fold molar excess of unlabelled M67 was added for 0, 5, and 10 min, respectively. **(B)** Specific DNA-binding activity was detected by autoradiography in vacuum-dried gels. **(C)** The histograms demonstrate the decline in specific DNA-binding activity during challenge with unlabelled M67 for STAT1-GFP (black columns) and untagged STAT1 (grey columns). Bars and asterisks indicate significant differences between samples over time.

In preliminary experiments, we assessed the DNA-binding activity of GFP-tagged and GFP-untagged recombinant STAT1 to a 43-mer DNA probe containing two high-affinity GAS sites with a spacing of 10 bp. As shown in Figure 2A, the two STAT1 proteins bound the probe, termed here 2xGAS, as dimeric and tetrameric complexes, indicating that either one or both of the tandem GAS sites were occupied. The high concentration of tyrosine-phosphorylated STAT1 in the EMSA reactions favours the formation of 2xGAS-bound tetramers over single dimers occupying only one GAS site and leaving the other unoccupied. The presence of a STAT1-specific antibody, but not an antibody directed against STAT3, significantly retarded the gel electrophoretic mobility of STAT1-containing complexes. As expected for intact cooperative, 2-site binding, competition with a 750-fold molar excess of unlabelled GAS oligonucleotides readily displaced STAT1 dimers occupying only one of the two tandem GAS sites, whereas binding of tetrameric complexes to two adjacent GAS binding sites was virtually unaffected (Figure 2A, lane 5).

Since we had previously shown that GFP tagging significantly reduces subcellular trafficking [28], we next wondered whether the time course of STAT1 release from a single consensus GAS site also differs between the GFP fusion and the untagged protein. For this purpose, we pre-incubated extracts from reconstituted, IFNγ-pre-treated U3A cells expressing either STAT1-GFP or untagged STAT1 with a [^33^P]-labelled M67 probe containing a single GAS site for 15 min on ice and subsequently added a 750-fold molar excess of unlabelled GAS elements for 0, 5 and 10 min, before the ice-cold reactions were loaded onto the gel (Figure 2B,C). Results from autoradiography showed that after 5 min of exposure on ice approximately two-thirds of the initial GAS-bound STAT1 dimers resisted the challenge with the high molar concentration of unlabelled DNA, and after an additional 5 min, DNA binding activity was further reduced (Figure 2B,C). However, we found that the dissociation kinetics from a single GAS sequence did not differ between the two STAT1 variants. Collectively, these data demonstrate that GAS binding is not affected by carboxyterminal fusion to GFP as the addition of the tag resulted in similar binding intensities and, furthermore, confirm the fast release of STAT1 dimers from a single GAS element.

### Binding and release of STAT1 from tandem GAS sites

**Figure 3:**
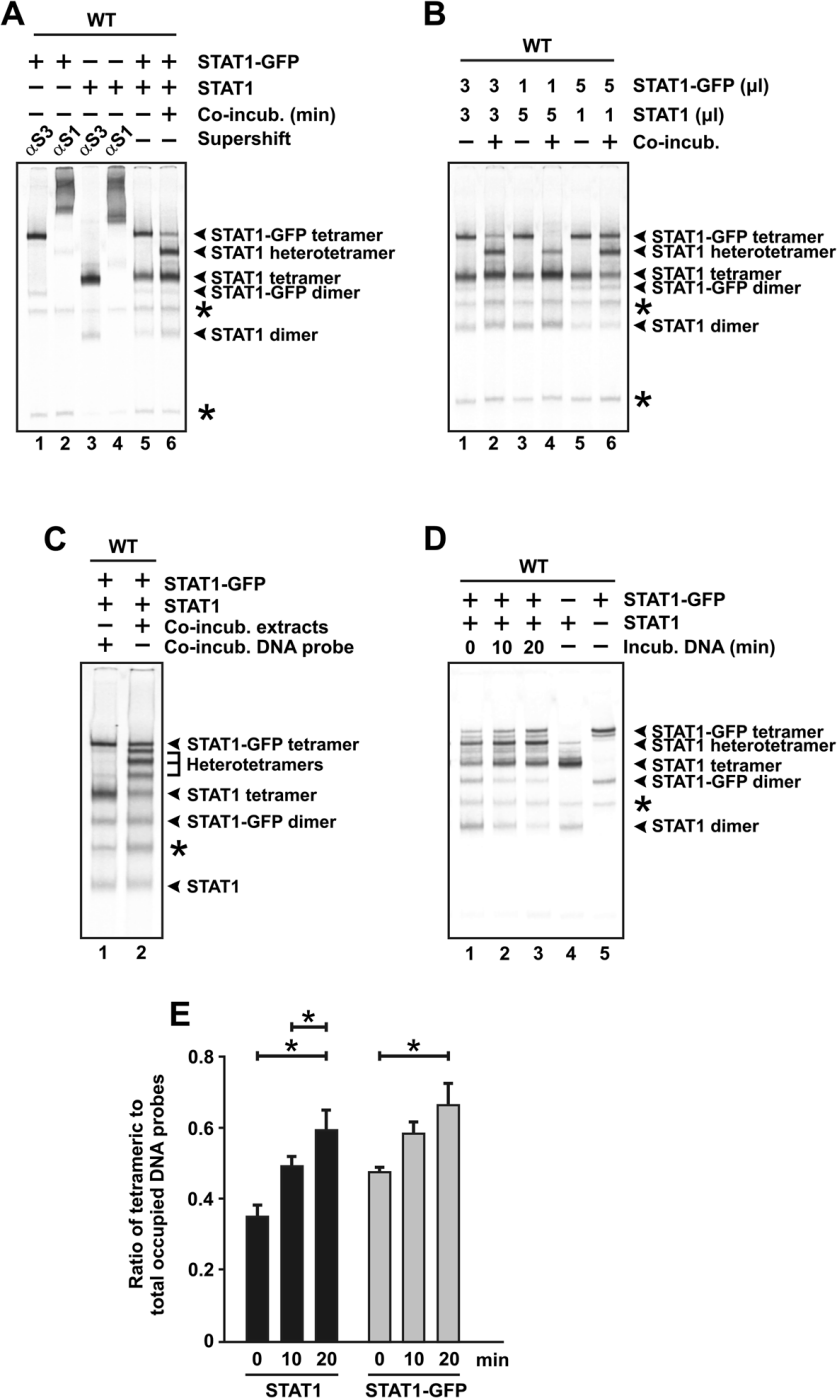
**(A) Demonstration of STAT1 heterotetrameric complexes occupying a double-stranded oligonucleotide containing two consensus GAS sites.** Cellular extracts from U3A cells expressing exclusively either GFP-tagged or untagged STAT1 were incubated separately with [^33^P]-labelled 2xGAS for 45 min (lanes 1 to 5) or, alternatively, mixed and co-incubated for 45 min (lane 6). Supershift reactions were performed by adding either an anti-STAT1 (lanes 2 and 4) or an unspecific anti-STAT3 antibody (lanes 1 and 3). Asterisks mark unspecific bands. **(B)** STAT1-GFP and STAT1 dimers compete for binding to 2xGAS. Different amounts of extracts from U3A cells expressing exclusively GFP-tagged or untagged STAT1 reacted either separately with [^33^P]-2xGAS or were mixed and reacted with the probe immediately before being loaded onto the gel. **(C)** Absence of binding to GAS elements promotes interdimeric protomer exchange. STAT1-GFP- and STAT1-containing extracts were co-incubated for 45 min in the presence (lane 1) or absence of [^33^P]-2xGAS (lane 2). Immediately before the reaction in lane 2 was loaded onto the gel, a similar amount of [^33^P]-2xGAS was added to the reaction as in lane 1. **(D)** Accumulation of interchanged STAT1 complexes at tandem GAS sites. Mixed STAT1-GFP- and STAT1-containing cellular extracts were incubated with [^33^P]-2xGAS for the indicated times, before being separated by gel electrophoresis (lanes 1 to 3). As controls, non-mixed extracts were used in lanes 4 and 5. **(E)** Time-dependent accumulation of tetrameric STAT1 at the expense of dimeric complexes. Histograms depict the ratio of tetrameric-to-total STAT1 binding activity presented as means and standard deviations from three independent experiments as shown in **(D)**. Bars and asterisks indicate significant differences in the pattern of tetrameric-to-total GAS occupancy.

Given that the DNA-binding properties are unaffected by the fusion with GFP, we next wondered whether the dissociation rate of STAT1 dimers into monomers exceeds that of dimer release from high-affinity GAS elements. To this end, we mixed equal amounts of tyrosine-phosphorylated GFP-tagged and untagged STAT1 together with [^33^P]-labelled 2xGAS and after 45 min of incubation loaded the reaction onto the gel. In parallel experiments, we added [^33^P]-2xGAS separately to each of the samples and only combined the two non-co-incubated reactions immediately before loading together into the same lane (Figure 3A). As expected, in the non-co-incubated EMSA reactions STAT1-GFP and untagged STAT1 migrated exclusively as two distinct bands corresponding to dimeric and tetrameric complexes, respectively. However, in the co-incubated reactions we noted the appearance of a new band which was absent in the non-co-incubated samples and migrated with a velocity that was intermediate between the two variants. This band corresponds to STAT1 tetramers consisting of a 1:1 stoichiometry of tagged and untagged STAT1 molecules, most likely resulting from the occupation of one STAT1-GFP homodimer and one adjacent untagged homodimer per duplex oligonucleotide.

To corroborate this observation, we varied the concentrations of tagged versus untagged STAT1 proteins and tested the co-incubated reactions for binding to [^33^P]-2xGAS (Figure 3B). The band intensity of the STAT1 variant with the lower concentration showed a steeper decline as compared to those with a higher concentration, indicating that it was dissipated as it contributed to the formation of a heterotetramer band. Notably, in the continuous presence of [^33^P]-2xGAS we did not observe the appearance of STAT1-GFP/STAT1 complexes composed of a 1:3 or 3:1 stoichiometry. Thus, as compared to the fast release of stable dimers from single GAS sites on ice, the dissociation of homodimers and formation of heterodimers at RT was not detectable under this reaction condition.

### GAS binding protects STAT1 dimers from protomer exchange

While the above-mentioned gel-shift reactions were performed in the continuous presence of radioactively labelled DNA, we also conducted experiments in which we first combined the GFP-tagged and GFP-untagged STAT1 variants for 30 min in the absence of GAS elements and added the DNA probe shortly before loading onto the gel. In controls, we incubated the extracts containing tagged and untagged STAT1 separately with [^33^P]-2xGAS for 30 min and then loaded the two reactions onto the same lane of the gel. This protocol was chosen to assess whether binding to high-affinity GAS sequences reduces the rate of protomer exchange. As seen in Figure 3C and D, in reactions allowing GFP-tagged and untagged homodimers to recombine in the absence of GAS sequences, there was a readily detectable pattern of newly formed bands corresponding to heterotetrameric complexes in 3:1, 1:1, and 1:3 stoichiometry, respectively. The assembly of asymmetric heterotetrameric complexes bound to 2xGAS was facilitated, when no GAS element was present in the reactions (Figure 3C, lane 2). Similarly, in the continuous presence of the GAS probe, we observed a time-dependent increase in the ratio of tetrameric-to-dimeric STAT1 complexes occupying double-stranded 2xGAS oligonucleotides. However, the presence of high-affinity DNA-binding sites during the entire incubation period significantly decreased the rate of heterotetramer formation (Figure 3D,E). Collectively, our data revealed the time-dependent *de novo* formation of STAT1-GFP/STAT1 heterodimers in various molar compositions which accumulated at tandem GAS binding sites as a consequence of an interdimeric exchange of protomers.

### Homotypic interactions between STAT1 N-domains are dispensable for the exchange of monomers

**Figure 4:**
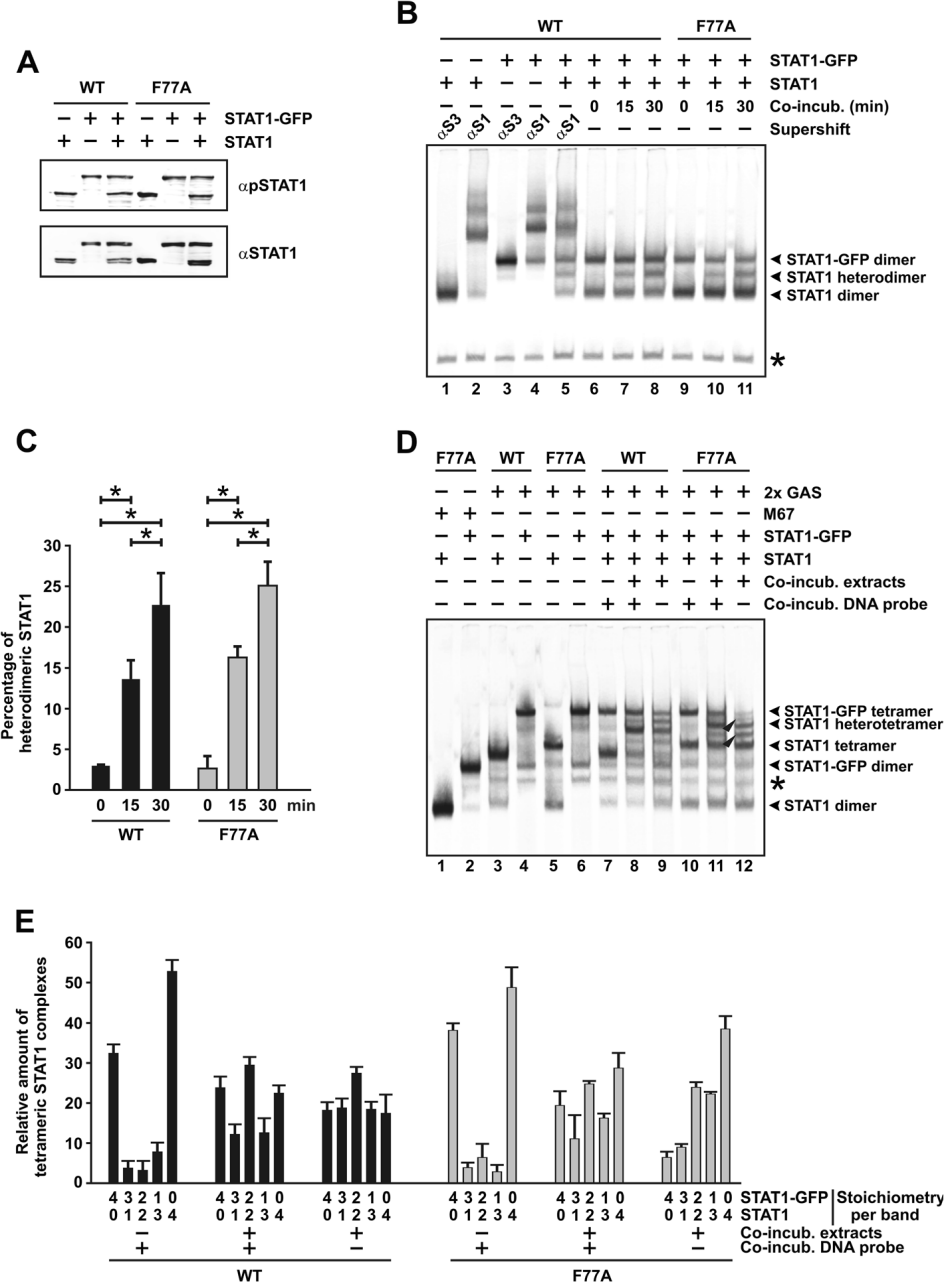
**Reciprocal aminoterminal interactions are not required for interdimeric protomer exchange. (A)** Expression of tyrosine-phosphorylated GFP-tagged and untagged STAT1 and their corresponding F77A mutants in cellular extracts used for EMSA. A representative Western blot experiment using a STAT1-specific phospho-tyrosine antibody (top panel) and the corresponding re-blot after the stripping off of bound immunoreactivity and re-incubation with pan-STAT1 antibody C-24 (bottom panel) is shown. **(B,C)** Mutation of phenylalanine 77 to alanine does not interfere with the formation of heterodimeric STAT1 complexes. Extracts from U3A cells expressing wild-type or mutant STAT1 with and without the GFP-tag were co-incubated and the occupancy of the M67 element by heterodimers monitored over time using EMSA. A typical autoradiogram **(B)** and a quantification of three similar experiments **(C)** are shown. The histograms present means and standard deviations as well as significant differences over time. **(D,E)** Aminoterminal contacts between monomers are dispensable for the dissociation and re-association of STAT1 dimers. Extracts from U3A cells expressing either STAT1-GFP or untagged STAT1 were separately incubated in the presence of [^33^P]-2xGAS before being loaded together onto the gel (lanes 7 and 10) or incubated as a mixture in the presence (lanes 8 and 11) or absence of [^33^P]-2xGAS, which in the latter case was added immediately before gel electrophoresis (lanes 9 and 12). Reaction time was 45 min for all samples. An asterisk at the gel margin marks an unspecific band. **(E)** Quantification of band intensities corresponding to tetrameric STAT1 bound to tandem GAS sites across the indicated stoichiometry of GFP-tagged versus untagged STAT1 molecules. Numbers under each column give the ratio of STAT1-GFP/STAT1 molecules for each band. The protocol used for these experiments was similar to that shown in **(D)**.

Mertens and colleagues proposed a model of conformational shift between parallel and antiparallel STAT1 dimer conformations in which reciprocal aminoterminal contacts are required to assist in freeing the phosphotyrosin-SH2 binding [22]. To test this hypothesis, we expressed the oligomerization-deficient point mutant F77A in STAT1-negative U3A cells and monitored the *de novo* formation of GAS-bound dimers in extracts of IFNγ-treated cells with time as compared to the wild-type protein. The phenylalanine residue in position 77 has been identified to mediate oligomerization of STAT1 dimers via aminoterminal-aminoterminal interactions between two adjacent monomers [26,29,30]. Given that upon stimulation of cells with an equal amount of IFNγ the pool of tyrosine-phosphorylated STAT1 molecules is significantly higher in the F77A mutant as compared to its wild-type counterpart [26], we first performed immunoblotting experiments to normalize phospho-protein concentrations allowing kinetic measurements in gel-shift assays (Figure 4A). Cellular extracts from IFNγ-stimulated cells containing similar amounts of phosphorylated STAT1-GFP and untagged STAT1 were then mixed and co-incubated for 0, 15 or 30 min at room temperature with [^33^P]-labelled M67 before being loaded onto a non-denaturing SDS gel. Autoradiograms demonstrated the appearance of a new band in reactions co-incubated for 15 min, which was absent when the extracts were incubated separately (Figure 4B). As compared to GFP-tagged and untagged homodimers, this newly formed band moved with an intermediate velocity and its intensity increased over 30 min of co-incubation (Figure 4B and C). Since the M67 probe can be occupied for spacing reasons only by a single STAT1 dimer, this band corresponds to newly formed STAT1-GFP/STAT1 heterodimers. Our results showed that the phenylalanine-to-alanine mutant displayed similar time kinetics for the *de novo* formation of STAT1 heterodimers as compared to the wild-type protein (Figure 4C). This finding demonstrated that aminoterminal-aminoterminal interactions are dispensable for the interdimeric exchange of protomers.

Next, we co-incubated extracts from cells expressing exclusively STAT1-F77A-GFP or STAT1-F77A in the presence of a DNA probe containing two high-affinity GAS elements for 30 min. Both mutant and wild-type STAT1 were able to be assembled into new heterotetramers (Figure 4D, lane 8 and 11, and Figure 4E). Notably, the concentrations of STAT1-GFP/STAT1 heterotetramers with a 3:1 and 1:3 stoichiometry were reduced in the presence of high-affinity GAS sites as compared to samples in which dimers were free to exchange their protomers due to the absence of DNA in the reactions (Figure 4D, compare lane 8 and 9, and Figure 4E). As judged from a slightly retarded electrophoretic mobility, the topology of tetrameric, but not dimeric complexes of mutant STAT1 bound to 2xGAS seemed to be somewhat less compact when compared to the wild-type protein, which in the case of the mutant most likely resulted from its lack of cooperative DNA binding [31]. In summary, we again found that GAS binding impaired the interdimeric exchange rate of protomers. Furthermore, we observed that substitution of alanine for the critical phenylalanine residue in position 77 did not affect the recombination of monomers between STAT1 dimers. We therefore concluded that homotypic interactions between STAT1 aminoterminal domains are not required for the process of protomer exchange.

## Discussion

In this paper, we describe a kinetic model of STAT1 protomer exchange which is based on the spontaneous dissociation of tyrosine-phosphorylated dimers into isolated monomers and their subsequent self-association (Figure 1). As compared to the fast release of STAT1 dimers from single GAS sites, the *in vitro* half-life of phosphorylated STAT1 dimers is relatively long and exceeds 10 min at room temperature. In addition, we found that binding to high-affinity GAS sites negatively affects the velocity of protomer exchange and thus critically decelerates the rate of the dissociation/re-association process. However, even in the absence of specific DNA-binding sites the dimeric structure of tyrosine-phosphorylated STAT1 remains considerably stable. Furthermore, we observed that the oligomerization-deficient point mutant STAT1-F77A displays similar kinetics of dimer dissociation as compared to the wild-type protein. Thus, in contrast to tetramer stabilization [26,30], we found no evidence for a role of a putative F77-mediated tether in facilitating the mutual transition between the two dimer conformations. This result demonstrates that reciprocal aminoterminal interactions between two adjacent monomers are dispensable for the formation of new dimer combinations.

Our findings provide support for the model proposed by Vinkemeier et al. that tyrosine phosphorylation regulates the partitioning of STAT1 between the parallel and anti-parallel dimer configuration [23]. The authors estimated that the half-life of the two STAT1 dimer conformations ranges between 20 and 40 min, which we confirm here for the phosphorylated dimer using a different technique [23]. From our study we cannot exclude the possibility that a constant oscillation between the two dimer configurations occurs at a much higher rate and that the critical F77 residue is engaged in this dynamic process. However, in this case, the proposed fast mutual interchange between the two dimer conformations cannot be the rate-limiting step for the dissociation and re-association of dimers and dimer stability should only marginally be affected by conformational flexibility.

The physiological significance of the observed interdimeric protomer exchange in terms of interferon-mediated signal transduction remains controversial. It is well-established that the antiparallel STAT1 dimer is the accessible substrate for the nuclear tyrosine phosphatase, while conversely DNA-binding protects parallel phospho-dimers from being tyrosine dephosphorylated [18,22]. The parallel-to-antiparallel reorientation of the phosphorylated STAT1 core allows for efficient dephosphorylation, as surface-exposed mutations in the interface destabilizing the antiparallel dimer conformation are associated with prolonged tyrosine phosphorylation levels [21,22]. Similarly, hyperphosphorylation was also observed for the homologous somatic L78R mutation in STAT3 found in inflammatory hepatocellular adenoma [32,33]. However, Domoszlai and co-workers reported that this mutation in the STAT3 molecule disturbs latent dimer formation and results in a monomeric population of unphosphorylated STAT3 [33].

Our kinetic measurements support the conclusion that the dissociation of STAT1 dimers into monomers may be the rate-limiting step in IFNγ-mediated signal transduction rather than the recognition of high-affinity GAS sites within the genome. Interestingly, a previous study using single-molecule microscopy has revealed numerous binding events of tyrosine-phosphorylated STAT1 in the nucleus indicative of distinct saltatory movements with residence times of up to 5 sec and intermittent diffusive motion [34]. This phenomenon was explained by the retardation and immobilization of activated STAT1 dimers when they randomly encounter their putative chromatin target sites. In line with the results from this single-molecule mobility analysis in living cells, our *in vitro* data confirm that the ephemeral binding to a single GAS site is substantially less stable than the long time scale dynamics of dimeric STAT1.

## Conclusions

In summary, our *in vitro* observations support the hypothesis that there is a constitutive dissociation of phospho-dimers into isolated monomers and their subsequent re-association into newly formed tyrosine-phosphorylated dimers. The EMSA experiments demonstrated that the interdimeric protomer exchange occurs at low rates when compared to the fast release from high-affinity GAS elements. Additionally, we found that binding to GAS sites has a negative impact on the protomer exchange rate by substantially impeding the recombination process of dimeric STAT1 in solution. However, reciprocal aminoterminal interactions between two adjacent partner monomers are not required for the dissociation and re-association of dimers, as determined by an unaltered interdimeric exchange rate of the oligomerization-deficient point mutant F77A as compared to its wild-type counterpart. Further investigations are required to elucidate the molecular basis by which the isolated STAT1 monomers recombine into newly formed dimers, which is of particular interest with respect to the regulation of gene activation in IFNγ-mediated signalling.

## Abbreviations

DTT: Dithiothreitol
EMSA: Electrophoretic mobility shift assay
GAS: Gamma-activated site
GFP: Green-fluorescent protein
IFNγ: Interferon-γ
JAK: Janus-activated kinase
NES: Nuclear export signal
SDS-PAGE: Sodium dodecyl sulfate-polyacrylamide gel electrophoresis
PCR: Polymerase chain reaction
RT-PCR: Reverse-transcriptase PCR
SH2 domain: Src-homology-2 domain
STAT1: Signal transducer and activator of transcription 1
WT: Wild-type
